# Enhancing Osseointegration of Zirconia Implants Using Calcium Phosphate Coatings: A Systematic Review

**DOI:** 10.3390/ma18194501

**Published:** 2025-09-27

**Authors:** Jacek Matys, Ryszard Rygus, Julia Kensy, Krystyna Okoniewska, Wojciech Zakrzewski, Agnieszka Kotela, Natalia Struzik, Hanna Gerber, Magdalena Fast, Maciej Dobrzyński

**Affiliations:** 1 Dental Surgery Department, Wroclaw Medical University, 50-425 Wroclaw, Poland; 2 Hospital in Swidnica, 58-100 Świdnica, Poland; ryszard.w.rygus@gmail.com; 3 Faculty of Dentistry, Wroclaw Medical University, 50-425 Wroclaw, Poland; julia.kensy@student.umw.edu.pl; 4 University Hospital in Wroclaw, 50-556 Wroclaw, Poland; krystyna.okoniewska@vet-farma.com.pl; 5 Medical Center of Innovation, Wroclaw Medical University, 50-425 Wroclaw, Poland; wojciech.zakrzewski1992@gmail.com (W.Z.); kotela.agnieszka@gmail.com (A.K.); 6 Pre-Clinical Research Centre, Wroclaw Medical University, 50-368 Wroclaw, Poland; 7 Department of Maxillofacial Surgery, Wroclaw Medical University, 50-556 Wroclaw, Poland; hanna.gerber@umw.edu.pl; 8 Department of Drug Form Technology, Wroclaw Medical University, 50-556 Wroclaw, Poland; magdalena.fast@umw.edu.pl; 9 Department of Pediatric Dentistry and Preclinical Dentistry, Wroclaw Medical University, 50-425 Wroclaw, Poland

**Keywords:** zirconia implant, CaP coating, osseointegration

## Abstract

Objective: Yttria-stabilized tetragonal zirconia polycrystal (Y-TZP), a variant of zirconia (ZrO_2_), has attracted interest as a substitute for titanium in dental and orthopedic implants, valued for its biocompatibility and aesthetics that resemble natural teeth. However, its bioinert surface limits osseointegration, making surface modifications such as calcium phosphate (CaP) coatings highly relevant. Materials and methods: The review process adhered to the PRISMA guidelines. Electronic searches of PubMed, Scopus, Web of Science, Embase, and Cochrane Library (July 2025) identified studies evaluating CaP-coated zirconia implants. Eligible studies included in vitro, in vivo, and preclinical investigations with a control group. Data on coating type, deposition method, and biological outcomes were extracted and analyzed. Results: A total of 27 studies were analyzed, featuring different calcium phosphate (CaP) coatings including β-tricalcium phosphate (β-TCP), hydroxyapatite (HA), octacalcium phosphate (OCP), and various composites. These coatings were applied using diverse techniques such as RF magnetron sputtering, sol–gel processing, biomimetic methods, and laser-based approaches. In multiple investigations, calcium phosphate coatings enhanced osteoblast attachment, proliferation, alkaline phosphatase (ALP) expression, and bone-to-implant contact (BIC) relative to unmodified zirconia surfaces. Multifunctional coatings incorporating growth factors, antibiotics, or nanoparticles showed additional benefits. Conclusion: CaP coatings enhance the bioactivity of zirconia implants and represent a promising strategy to overcome their inertness. Further standardized approaches and long-term studies are essential to verify their translational relevance.

## 1. Introduction

**Figure 1:**
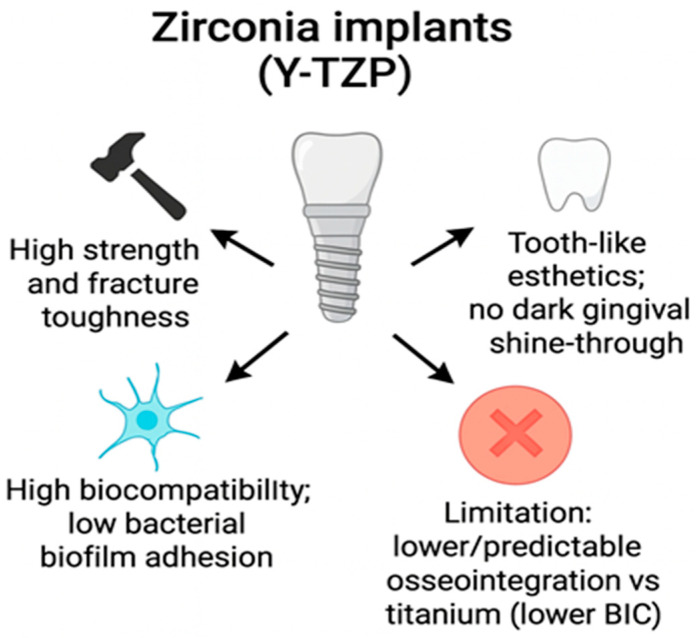
Key properties of zirconia (Y-TZP) implants.

Among ceramic biomaterials, yttria-stabilized tetragonal zirconia polycrystal (Y-TZP) has been gaining more attention as a substitute to titanium in implantology, as it exhibits favorable mechanical and biological properties. The high flexural strength, fracture resilience, and hardness of Y-TZP ensure sufficient resistance to biomechanical loads in the oral environment [1,2]. Its tooth-like white color provides a significant esthetic advantage over titanium, especially in the anterior maxilla, where gingival transparency may compromise visual outcomes [3]. Beyond dentistry, zirconia ceramics have also been investigated in orthopedic applications, including knee and hip prostheses, owing to their resistance to wear and low friction coefficient [4,5]. Zirconia demonstrates favorable biological properties, including high biocompatibility, low allergic potential, and diminished bacterial biofilm adherence, which may decrease the risk of peri-implantitis [6,7]. Despite these advantages, the osseointegration capability of zirconia implants has been reported to be inferior or less predictable in comparison with titanium implants [8]. Preclinical studies show that zirconia can achieve comparable bone-to-implant contact (BIC) to titanium after appropriate surface modification, but clinical performance is still less consistent [9]. Nevertheless, it is crucial to emphasize that although zirconia demonstrates excellent mechanical strength, high biocompatibility, and favorable esthetic properties, its surface is considered relatively bioinert. Unlike titanium, which naturally forms a stable oxide layer supporting direct bone bonding, untreated zirconia exhibits lower protein adsorption and reduced cellular attachment, resulting in less predictable osseointegration. This intrinsic limitation provides a strong rationale for surface modification. In particular, calcium phosphate coatings are of great interest because they not only counteract zirconia’s bioinert character but also create a bioactive interface that resembles natural bone mineral, thereby enhancing early bone formation and long-term stability of the implant. Therefore, various strategies of surface modifications have been extensively explored to improve the bioactivity of zirconia [10,11]. Among these approaches, calcium phosphate (CaP) coatings are especially promising, as they replicate the inorganic bone phase and markedly enhance osteoconduction in both dental and orthopedic applications [12,13] (Figure 1).

**Figure 2:**
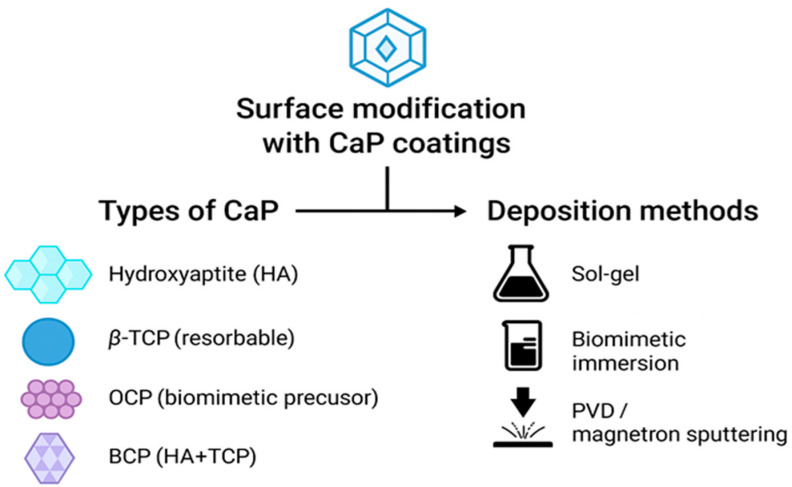
Schematic illustration of the surface alteration of zirconia implants with calcium phosphate (CaP) coatings.

Calcium phosphate ceramics, including β-tricalcium phosphate (β-TCP), hydroxyapatite (HA) and octacalcium phosphate (OCP), are widely acknowledged for their chemical resemblance to bone mineral composition and their capacity to enhance osseointegration [14]. HA is the most extensively studied, providing excellent osteoconductivity, strong bioactivity, and favorable cell adhesion properties [15]. β-TCP, in contrast, is more resorbable, promoting dynamic bone remodeling [16,17]. OCP is considered a precursor phase in biomineralization and has been shown to rapidly nucleate and integrate onto zirconia substrates, offering strong interfacial bonding [18,19]. Biphasic calcium phosphate (BCP) which is a mixture of HA and TCP, balances stability and resorption, supporting controlled bone remodeling [20]. CaP coatings are also employed in orthopedic implants (e.g., hip stems, spinal cages) to promote early fixation and long-term integration in load-bearing sites [21]. Ion-substituted CaPs (Mg^2+^, Sr^2+^, Zn^2+^) further enhance osteoblast proliferation and differentiation while imparting antibacterial effects [22,23]. Thus, CaP coatings provide not only a bioactive interface but also versatile functionalities, expanding their potential use from dentistry to orthopedic implantology (Figure 2).

Multiple deposition techniques have been developed for applying CaP coatings onto zirconia implants, each influencing morphology, roughness, crystallinity, and adhesion strength. The sol–gel method enables the fabrication of thin, uniform HA layers with high adhesion and tunable porosity [24]. Biomimetic deposition replicates natural bone mineralization, producing OCP and BCP coatings with bioactive properties [9]. Physical vapor deposition (PVD) and magnetron sputtering allow precise control of thickness and composition [25]. Laser-assisted modifications (Nd:YAG, CO_2_) increase surface wettability and roughness, thereby improving CaP adhesion and cellular interactions [26,27]. Hybrid approaches combining mechanical surface texturing with biomimetic deposition have improved coating durability under cyclic loading [28]. Such strategies not only improve performance in dentistry but are also relevant for orthopedic load-bearing implants, where coating adhesion and mechanical reliability are critical (see Figure 2).

CaP-coated zirconia implants have shown significant biological benefits in vivo and in vitro. HA- or OCP-coated surfaces enhance the activity of alkaline phosphatase (ALP), osteoblast proliferation, and the expression of osteogenic genes compared with uncoated zirconia [29]. Ion-doped coatings (Mg, Sr) further accelerate osteoblast differentiation and mineralization while potentially reducing peri-implant bacterial colonization [30]. In vivo animal studies confirm significantly higher BIC values for CaP-coated zirconia compared with uncoated implants, reaching levels similar to titanium [15]. Morphology strongly influences outcomes, as porous or nanostructured CaP coatings promote early bone apposition and vascular infiltration [22,31]. In orthopedics, similar trends are reported, where CaP-coated zirconia and other ceramics support stable fixation and reduce micromotion at the bone–implant interface [32,33]. These findings indicate that CaP coatings can overcome the bioinert nature of zirconia and extend its applicability across both dental and orthopedic implantology.

Several systematic reviews have discussed calcium phosphate coatings in the context of bone implants. Most of these works, however, focused primarily on titanium, which remains the clinical gold standard, and mentioned zirconia only briefly or grouped it together with other ceramic substrates [34,35]. For example, Bral et al. [34] provided a comprehensive overview of CaP-coated titanium implants, emphasizing their clinical success, while López-Valverde et al. [35] summarized general surface modification strategies for ceramics without a zirconia-specific analysis. This review seeks to thoroughly assess the latest evidence concerning the impact of calcium phosphate coatings on the osseointegration of zirconia implants. It encompasses studies from the past two decades across dental and orthopedic applications, including in vivo, in vitro, and preliminary clinical investigations. Attention is given to the type of CaP used (HA, β-TCP, OCP), deposition methods, and biological outcomes (cell proliferation, ALP activity, BIC values). To date, no comprehensive review has synthesized these aspects into a unified perspective. By addressing this gap, our work provides a foundation for future translational research and highlights the potential of CaP-coated zirconia implants as bioactive solutions for both dentistry and orthopedics.

## 2. Materials and Methods

### 2.1. Focused Question

The systematic review adhered the PICO framework [36] in the following manner: In patients receiving zirconia dental implants (P), does the addition of a calcium phosphate coating (I), compared with uncoated zirconia implants (C), result in improved osseointegration (O)?

### 2.2. Protocol

**Figure 3:**
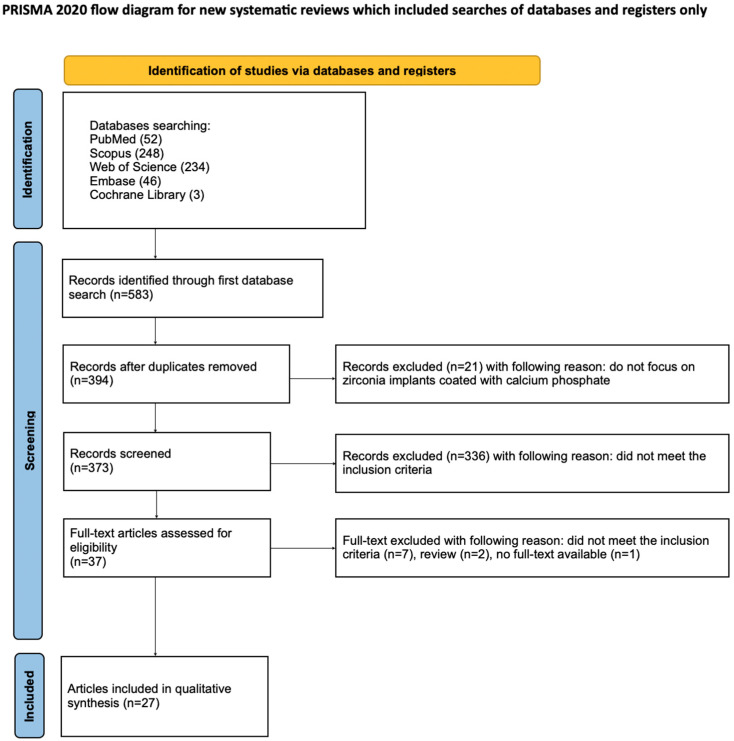
The PRISMA 2020 flow diagram [37].

The process of study selection was presented using a PRISMA flow diagram (Figure 3) [37]. This systematic review was conducted in accordance with the Preferred Reporting Items for Systematic Reviews and Meta-Analyses (PRISMA) Statement [37]. In addition, the review was preregistrated on the Open Science Framework (OSF) at the provided link: https://osf.io/54pcx (accessed on 22 August 2025).

### 2.3. Eligibility Criteria

Eligibility for inclusion was determined based on the following criteria [38,39,40,41,42,43,44]:

- Use of zirconia orthopedic or dental implant;
- Calcium phosphate coated implants;
- In vivo and in vitro studies;
- Presence of a control group;
- Prospective case series;
- Randomized controlled clinical trials (RCT);
- Non-randomized controlled clinical trials (NRS);
- Papers in English.

Studies were excluded if they fulfilled any of the subsequent criteria, as determined by the reviewers [38,39,40,41,42,43,44]:

- Other than zirconia implants used;
- No CaP coating;
- Non-English papers;
- Duplicated publications;
- Opinions;
- Editorial papers;
- Review articles;
- Clinical reports;
- No access to full-text versions.

No limitations were imposed concerning the year of publication.

### 2.4. Information Sources, Search Strategy and Study Selection

An electronic search was carried out in July 2025 across five major databases: PubMed, Scopus, Web of Science (WoS), Embase, and the Cochrane Library. For each source, database-specific syntax was applied to capture studies concerning zirconia implants coated with calcium phosphate.

PubMed: The query combined title and abstract terms:

(“calcium phosphate”[Title/Abstract] OR “calcium phosphates”[Title/Abstract] OR TCP[Title/Abstract] OR “tricalcium phosphate”[Title/Abstract] OR β-TCP[Title/Abstract]) AND (zirconia[Title/Abstract] OR zirconium[Title/Abstract]) AND (implant[Title/Abstract] OR implants[Title/Abstract]).

Scopus: The search string was restricted to titles, abstracts, and keywords:

TITLE-ABS-KEY(“calcium phosphate” OR “calcium phosphates” OR TCP OR “tricalcium phosphate” OR β-TCP) AND TITLE-ABS-KEY(zirconia OR zirconium) AND TITLE-ABS-KEY(implant OR implants).

Web of Science (WoS): The Topic Search (TS) field was used:

TS = (“calcium phosphate” OR “calcium phosphates” OR TCP OR “tricalcium phosphate” OR β-TCP) AND TS = (zirconia OR zirconium) AND TS = (implant OR implants).

Embase: Terms were restricted to title and abstract fields:

(‘calcium phosphate’:ti,ab OR ‘calcium phosphates’:ti,ab OR TCP:ti,ab OR ‘tricalcium phosphate’:ti,ab OR β-TCP:ti,ab) AND (zirconia:ti,ab OR zirconium:ti,ab) AND (implant:ti,ab OR implants:ti,ab).

Cochrane Library: A simplified syntax was applied:

(“calcium phosphate” OR “tricalcium phosphate” OR TCP OR β-TCP) AND (zirconia OR zirconium) AND (implant OR implants).

Only peer-reviewed full-text articles meeting the eligibility criteria were chosen for additional analysis.

### 2.5. Data Collection Process and Data Items

Five researchers (A.K., J.K., W.Z., K.O., N.S.) separately performed the screening and data extraction. For each eligible study, information was gathered regarding the first author, article title, study design, year of publication, type of laser used, and the reported outcomes of osseointegration. All extracted data were organized in a standardized Excel spreadsheet to ensure consistency and traceability.

### 2.6. Risk of Bias and Quality Assessment

In the preliminary initial screening stage, two reviewers evaluated the titles and abstracts independently to reduce possible bias. The level of concordance between them was quantified using Cohen’s kappa (κ) statistic. Any differences in study selection were discussed and resolved until consensus was reached [45].

### 2.7. Quality Assessment

Two reviewers (M.D. and J.M.), blinded to each other’s assessments, assessed the methodological quality of the selected studies utilizing the Joanna Briggs Institute (JBI) checklist for quasi-experimental (non-randomized) designs [41]. Every item on the checklist was evaluated using four possible responses: “yes,” “no,” “unclear,” or “not applicable.” Discrepancies between reviewers were resolved through discussion until consensus was achieved. The level of inter-rater agreement was evaluated with Cohen’s kappa, computed using MedCalc software (version 23.1.7, MedCalc Software Ltd., Ostend, Belgium). The resulting κ value of 0.82 (*p* < 0.001) reflected an excellent level of reliability and consistency among the reviewers.

## 3. Results

### 3.1. Study Selection

**Figure 4:**
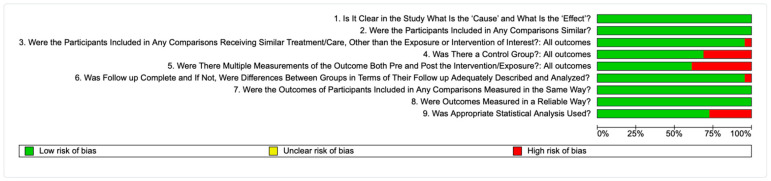
Risk of bias of included studies.

Figure 4 shows the study selection procedure according to the PRISMA guidelines. The preliminary search of Scopus, PubMed and WoS yielded 394 possibly relevant records. At this stage, a number of articles rejected for the following reasons: many papers investigated titanium implants rather than zirconia; others did not involve surface functionalization with calcium phosphate coatings; and additional papers were reviews, case reports, editorials, or conference abstracts rather than original research. Only full-text research articles written in English and focusing on zirconia dental implants with calcium phosphate functionalization were considered eligible for inclusion. During the titles and abstracts screening, 367 papers were excluded for failing to meet the criterion of inclusion, leaving 27 studies for full evaluation. Ultimately, these 27 articles were evaluated in the review. The considerable heterogeneity of the included studies prevented the conduct of a meta-analysis.

### 3.2. General Characteristics of Included Studies

In total, twenty-seven studies followed the inclusion criteria for this systematic review. Their key characteristics are listed in Table 1. The central research question addressed was whether calcium phosphate coatings on zirconia implants contribute to improved osseointegration.

Several investigations focused on developing and optimizing coating techniques, like laser-assisted β-TCP deposition [46,47], RF-magnetron sputtering [48,49], wet powder spraying [50,51], biomimetic methods [52,53,54], and ion beam-assisted deposition [18,55,56]. Others explored the incorporation of hydroxyapatite using aerosol deposition [57,58], functional biomolecules such as BMP-2 [59] or FGF-2 with low molecular weight heparin [19], and antimicrobial or drug-loaded nanoparticles [54,60].

A high number of studies combined physicochemical and mechanical analyses with in vitro evaluations of osteoblast adhesion, proliferation, and differentiation [18,48,50,51,56,57,61,62,63,64], while others additionally performed in vivo assessments in animal models, mainly dogs, rabbits, and rats [49,53,59,65,66]. The early contact between bone and implant, as well as the bone formation showed to be significantly improved in several in vivo studies using hydroxyapatite or calcium phosphate-modified zirconia implants [18,53,66].

Some authors aimed to create multifunctional or hybrid coatings, introducing silver nanoparticles for antibacterial activity [60], antibiotic-loaded biomimetic CaP layers [54], or biohybrid systems combining β-TCP coatings with stem cell layers to regenerate periodontal ligament structures [47]. Other approaches involved surface modifications at the nanoscale, such as mesoporous zirconia nanocapsules for biomineralization precursor delivery [67] or amino acid–based chemical grafting to promote calcium phosphate nucleation [64].

**Table 1:** General Characteristics of Included Studies.

Study	Aim of the Study	Material and Methods	Results	Conclusions
Safi [46]	Evaluation of β-TCP coating applied to zirconia substrate using Nd:YAG laser as a potential method for functionalizing dental implants.	β-tricalcium phosphate (β-TCP) layer applied to zirconia using direct laser melting. Analyses: - Field emission scanning electron microscope (FESEM), - Energy Dispersive X-ray (EDX) line scan, - X-ray diffraction (XRD), -Vickers hardness, -nanoindentation (elastic modulus).	-~27 μm thick coating with good adhesion and dual-layer structure (1–2 μm dense + 25 μm porous). -β-TCP confirmed with proper chemical composition and Ca/P ratio. -Hardness 545–690 HV, elastic modulus matched natural bone.	Laser-applied β-TCP on zirconia shows good adhesion, biocompatibility, and mechanical properties suitable for dental implants. Laser melting is an effective zirconia implant functionalization method.
Safi [47]	Development and evaluation of biohybrid dental implants coated with β-TCP (on zirconia and titanium) integrated with stem cell layers to regenerate periodontal ligament (PDL).	Zirconia and titanium implants coated with β-TCP via Nd:YAG laser. Periodontal Ligament Stem Cells (PDLSCs) and Bone Marrow Mesenchymal Cells (BMMSCs) from rabbits cultured separately and in co-culture, applied to implants and implanted in rabbit mandibles for 45 or 90 days. Microscopy: -H&E, -FESEM, -immunofluorescence; -evaluated periostin expression, PDL width, and fiber attachment.	-β-TCP-only implants integrated well with bone but did not form PDL-like tissue. -Implants with PDLSC or co-cultures formed PDL-like tissue with periostin expression. Co-cultures were most effective. -No difference between titanium and zirconia.	Zirconia implants with β-TCP and engineered stem cell layers can regenerate PDL, forming biohybrid implants. PDLSC and BMMSC co-cultures were the most effective, suggesting potential for next-gen PDL-functional implants.
Kozelskaya [48]	Comparison of biological, mechanical physicochemical, properties of Ca-P coatings applied to ZrO_2_ substrates via RF magnetron sputtering using various Ca-P powders.	Yttria-stabilized ZrO_2_ ceramic plates coated with 5 Ca-P powders: -calcium phosphate tribasic (CPT), -HA, monophosphate, -calcium phosphate dibasic dehydrate (DCPD), -calcium pyrophosphate powder (CPP). Characterized for: -morphology, -thickness, -hardness, -chemical composition, -MSC adhesion/survival. Tools: -AFM, -SEM-EDX, -nanoindentation, -FTIR, -wettability, -fluorescence.	-Best MSC adhesion and coverage with CPT coating, (closest mechanically to ZrO_2_) -All coatings amorphous but differed in surface properties. -HA most hydrophilic, CPT most hydrophobic. -No cytotoxicity observed.	CPT is the most promising for future ceramic dental implant research. In vivo studies are required to confirm osteoinductive potential.
Pardun [50]	Assessment of the impact of mixed zirconia–calcium phosphate (TZ-CP) coatings on dental implants regarding coating stability, mechanical strength, and bioactivity.	Zirconia discs and implants coated with different TZ:CP ratios using wet powder spraying. Analyses: -SEM, -XRD, -roughness, -B3B test, -scratch resistance, -Ca^2+^ release, -SBF immersion. Cell studies: -osteoblast proliferation, -morphology, -ALP activity.	-Higher TZ content increased coating stability and strength; -higher CP increased bioactivity and Ca^2+^ release. 1:1 and 2:1 TZ:CP ratios had best balance. -Better osteoblast proliferation with more TZ.	TZ-CP coatings offered a tunable balance between bioactivity and mechanical stability. Ratios of 1:1 and 2:1 showed optimal outcomes for dental implants with good adhesion, cell response, and osseointegration.
Stefanic [52]	Development of a simple, fast biomimetic method for depositing octacalcium phosphate (OCP) coatings on zirconia (Y-TZP) to enhance bioactivity for implant use in dentistry.	Two-step biomimetic coating using CPS1 (pH 7.4) and CPS2 (pH 7.0). Step 1: soak in CPS1 (1 h) to form thin Ca-P layer. Step 2: soak in CPS2 (1–24 h) for thick OCP layer. Characterization: -SEM, -TEM, -XRD, -FTIR, -NGIA, -AFM, -profilometry, -adhesion test.	-First step formed thin (~200 nm) Ca-P layer. Second step gave continuous ~5 µm lamellar OCP coating. -Strong adhesion (94–95% retention; ASTM 4B). -Coatings contained CDHA and OCP phases.	A reproducible, fast method was developed for OCP coatings that are uniform, stable, and suitable for dental implants. Thickness can be controlled, and method allows future bioactive molecule incorporation.
Pardun [51]	Development and characterization of a mixed calcium phosphate and zirconia oxide (TZCP) coating applied via wet powder spraying (WPS) on zirconia implants to enhance bioactivity and mechanical strength.	CP, TZ, and TZCP coatings applied via WPS, sintered at 1500 °C. Characterization: -SEM, -EDX, -AFM, -GI-XRD, -profilometry, -scratch and bone tests, -B3B bending test, -EDX elemental mapping. In vitro tests on bovine bone and polyurethane foam.	-TZCP had uniform, porous (~17%) coatings with ~4 µm roughness and no cracks. -CP had cracks, low adhesion, and damage. -Only TZ and TZCP passed scratch tests. -TZCP had strength similar to uncoated zirconia (~1023 MPa). -TZCP maintained integrity after implantation; CP failed.	TZCP showed more effective adhesion and stability than CP and similar to TZ. WPS allows coatings on complex zirconia implant shapes with good quality. In vivo studies needed to confirm bioactivity.
AlFarraj [65]	Evaluation of bone contact with zirconia and titanium implants, with or without hydroxyapatite (HA) coating.	32 implants (16 Ti, 16 Zr; half with HA) implanted in 16 rabbits. After 8 weeks, samples analyzed histologically and histomorphometrically for bone-implant contact (BIC).	Mean BIC: -Zr—45.1%, -Ti—45.5%, -Zr + HA—60.3%, -Ti + HA—59.8%. No inflammation, good integration across all groups.	Zirconia and titanium implants show similar bone integration. HA coating did not provide significant benefit in the 8-week rabbit model.
Yang [68]	Development of a high-strength, multilayered hydroxyapatite (HA)/tri-calcium phosphate (TCP) coating on porous zirconia substrates to create load-bearing, osseointegrative scaffolds suitable for bone repair implants.	A zirconia core enriched with HA was presintered to form microporosity. Three HA-based coating layers -pure HA, -HA with alumina/zirconia, -transitional HA-zirconia were applied using slip-coating and co-sintered at 1300 °C. Characterization: -mechanical testing, -SEM/FIB imaging, -in vitro biocompatibility assays with L929 fibroblasts	The composite showed high bending strength (321 MPa) and strong coating adhesion (~24.5 MPa). The coating featured interconnected pores (1–50 µm) and excellent interfacial bonding. Cell viability exceeded 90%, and SEM confirmed good cell adhesion and morphology.	The layered HA/TCP-zirconia scaffold offers a promising solution for load-bearing implants, with excellent mechanical performance and biocompatibility.
Kong [61]	Evaluation of zirconia–alumina (ZA) nanocomposites modified with hydroxyapatite (HA) to enhance biocompatibility while maintaining high mechanical strength for use in load-bearing dental or orthopedic implants.	ZA nano-composite powder (80% ZrO_2_, 20% Al_2_O_3_) was synthesized via the Pechini process. Composites containing up to 40 vol% HA were fabricated by hot-pressing at 1400 °C. Characterization: -flexural strength (four-point bending) -proliferation of MG63 and HOS -alkaline phosphatase (ALP) activity in vitro.	Mechanical: Flexural strength decreased with increasing HA content but remained high (e.g., 1270 MPa at 5% HA). Structural: Biphasic calcium phosphate (HA/TCP) formed during sintering; uniform microstructure retained. Biological: Both cell proliferation and ALP activity showed significant elevation with HA content. At 40% HA, both parameters were comparable to pure HA, indicating excellent biocompatibility.	Promising balance between strength and bioactivity. A 30% HA content was optimal for applications requiring both mechanical support and enhanced osseointegration.
Lee [57]	Assessment of hydroxyapatite (HA) coating on 3Y-TZP zirconia using aerosol deposition (AD) to improve surface bioactivity without compromising mechanical properties.	HA powder was deposited onto zirconia disks using the AD technique at room temperature. Characterization: -surface roughness, -morphology (SEM), -crystal structure (XRD), -adhesive strength, -adhesion of MG63 cells -proliferation of MG63 cells, -ALP activity.	Surface: Coated zirconia had rougher surfaces with evenly distributed HA particles and maintained crystalline HA structure. Adhesion: Coating adhesion strength reached ~21 MPa. Biological: Enhanced MG63 cell adhesion, proliferation, and ALP expression were observed compared to uncoated zirconia.	Aerosol-deposited HA coatings improve zirconia bioactivity while preserving mechanical properties. The method provides a viable approach for enhancing osseointegration of zirconia-based implants.
Ferguson [55]	To assess the in vitro and in vivo performance of calcium phosphate–coated zirconia implants produced by ion beam-assisted deposition (IBAD).	Zirconia implants were coated with calcium phosphate using IBAD, which creates a dense, nanocrystalline layer. Coated and untreated implants were evaluated for: In vitro: -surface properties, -crystallinity, -MG63 cell proliferation. In vivo: -implants were applied into rat femurs and assessed after 4 and 12 weeks using histology and histomorphometry.	Surface: IBAD created a uniform, nanostructured coating without cracking or delamination. In vitro: Coated implants significantly enhanced MG63 cell proliferation. In vivo: Coated implants showed better bone-to-implant contact (BIC) at both time points, with more rapid and extensive new bone formation than controls.	IBAD calcium phosphate coatings improved both early and long-term osseointegration of zirconia implants, making them a promising option for load-bearing dental and orthopedic applications.
Kim [62]	Development of calcium phosphate-coated porous zirconia scaffolds with controlled dissolution rates to enhance osteoblast differentiation and osseointegration.	Porous zirconia samples were produced using a polyurethane foam template and coated with five types of calcium phosphate: -hydroxyapatite (HA), - tricalcium phosphate (TCP), -fluorapatite (FA), - HA + TCP, - HA + FA. An intermediate FA layer was used to stabilize HA during sintering. Coated scaffolds were heat-treated. Characterization: - SEM - XRD - Ca^2+^ release, MG63 and HOS cell culture assessment: -morphology, -proliferation, -ALP activity	All coatings uniformly applied—XRD confirmed structural stability, with the FA layer preventing phase transformation. Dissolution rates varied by composition: -The highest in case of TCP -The lowest in case of FA. HA-containing coatings (especially HA + FA and HA + TCP) marked elevated ALP activity, suggesting improved osteoblastic differentiation Cell proliferation comparable across all groups.	New coatings provide both structural integrity and biological activity. Coatings with HA and FA balance dissolution and bioactivity, supporting their use in bone-regenerative applications requiring gradual ion release and enhanced cell differentiation.
Langhoff [66]	Evaluation of osseointegration and early bone reaction to zirconia implants coated with calcium phosphate (CaP) in a preclinical canine model.	Zirconia implants were coated with a 15 µm thick layer of calcium phosphate using a low-temperature coating process and then inserted into the mandibles of six dogs—each received both coated and uncoated zirconia implants in a split-mouth design. Histological analysis was conducted 1, 2, and 4 weeks after implantation to assess: - bone formation; -bone-to-implant contact (BIC).	CaP-coated implants demonstrated a more rapid bone apposition in early stages. At 1 week, coated implants exhibited new bone trabeculae directly attached to the surface. BIC was significantly higher in coated implants at week 1, but differences decreased over time.	The application of calcium phosphate coating on zirconia implants has the potential to enhance the initial phases of osseointegration. Surface modification may be advantageous for enhancing initial bone healing and implant stability.
Cheng [18]	To develop and optimize calcium phosphate coatings on zirconia using ion beam-assisted deposition, with the goal of enhancing surface bioactivity and improving osseointegration	HA coatings were applied to Y-TZP zirconia substrates using ion beam-assisted deposition at low temperature. Characterization: -SEM, -EDS, -XRD, - scratch testing (adhesive strength) MG63 cell culture: -adhesion, -proliferation, -morphology.	The IBAD process produced a uniform, nanocrystalline HA coating (~2 µm thick) without cracks or delamination, with good adhesion and retention of HA crystalline structure. Cell studies showed significantly enhanced adhesion, proliferation, and spreading on coated surfaces compared to bare zirconia. SEM confirmed well-attached, polygonal cells with filopodia on HA-coated samples.	IBAD allows for stable deposition of bioactive HA on zirconia at low temperatures, improving surface characteristics and cellular responses without compromising mechanical properties.
Mutsuzaki [49]	Evaluation of osseointegration and bone remodeling around hydroxyapatite-coated zirconia implants using a novel RF-magnetron sputtering technique in a rabbit femur model.	HA coatings (approximately 1 µm thick) were applied to zirconia implants using RF-magnetron sputtering. Coated and uncoated implants were placed into rabbit femurs. After 2 and 4 weeks, samples were harvested for: -histological analysis -histomorphometry to assess the contact between bone and implant (BIC) and bone area (BA) of osseointegration.	-direct bone apposition on both coated and uncoated implants -BIC and BA values notably elevated in the HA-coated group at both time intervals -At 4 weeks, coated implants demonstrated improved new bone formation, denser trabecular structure, and closer bone contact than uncoated controls.	HA coating via RF-magnetron sputtering enhances early osseointegration of zirconia implants by promoting faster and more robust bone response.
Cruz [53]	Investigation of biomimetic calcium phosphate coatings on zirconia implants and their in vivo effect on bone integration in a canine model.	Zirconia implants were treated with a two-step biomimetic process to form a calcium phosphate coating using simulated body fluid. Treated and untreated implants were inserted into the mandibles of six dogs and analyzed after 1 and 6 weeks. Characterization: -histological -histomorphometric to assess bone-to-implant contact (BIC) and bone area fraction (BAF).	-greater BIC and BAF values compared to uncoated controls. -at 1 week, the coated implants showed earlier bone formation and higher density of new bone matrix -at 6 weeks, the difference in BIC was still evident	Biomimetic calcium phosphate coatings significantly enhance early bone reaction to zirconia implants.
Goldschmidt [60]	To develop a dual-function coating for zirconia dental implants by incorporating silver nanoparticles into calcium phosphate layers to enhance antibacterial effect and bone integration.	Zirconia discs were sintered at 1450 °C, sandblasted, coated using a two-stage process: 1. calcium phosphate pre-coating in 2× concentrated SBF (3 days, 40 °C), 2. silver nanoparticle incorporation (40–60 nm, 0.1–3.0 g/L) in fresh SBF (3 days, 40 °C). Characterization: -SEM-EDX -XRD, -cytotoxicity tests with MG63 cells -antibacterial efficacy against *S. aureus* and *E. coli.*	Silver incorporation depended on concentration and positioning, with horizontal samples showing higher silver content. Only samples with 0.05% silver (0.1 g/L vertical positioning) were cytocompatible in direct contact. Higher silver concentrations had better antimicrobial activity against *S. aureus* but increased cytotoxicity. *E. coli* growth was inhibited on all surfaces.	Biomimetic precipitation created zirconia coating with calcium phosphate bioactivity and silver antimicrobial properties. 0.05% silver content achieved both cytocompatibility and bacterial inhibition.
Yasuaga [69]	To increase the biological activity of fibroblast growth factor 2 (FGF-2) in calcium phosphate coatings by adding low molecular weight heparin (LMWH) to improve implant surface for orthopedic and dental applications.	Zirconia substrates were sintered at 1350 °C, followed by polishing and, immersion coating in supersaturated calcium phosphate solution containing LMWH (0.04–4 IU/mL) and FGF-2 (4 μg/mL) at 37 °C for 24 h. Characterization: -SEM-EDX -XRD, -cell proliferation (NIH3T3, HUVECs), -tube formation assays, -osteogenic gene expression (BMP-2, RUNX2, COLIA1).	LMWH incorporation was dose-dependent and significantly increased FGF-2 content and stability in the coatings. LMWH-FGF-2 composite layers showed enhanced mitogenic activity in both cell types, improved tube formation with increased length and branch points, and upregulated osteogenic markers (BMP-2, RUNX2) while downregulating COLIA1.	LMWH enhanced FGF-2 biological activity by improving stability and incorporation, creating multifunctional coatings with enhanced mitogenic, angiogenic, and osteogenic properties for implant applications.
Huang [67]	Development of a delivery system for nanocapsules made of porous zirconium oxide with a mesoporous structure, used for the prolonged release.	Hollow mesoporous zirconia nanocapsules synthesized using a hard template method with silica particles, zirconia coating, calcination at 850 °C. Polyallylamine-stabilized amorphous calcium phosphate precursors were loaded via electrostatic interactions. Characterization: -TEM, -STEM-EDX, -XRD, -FTIR, -porosimetry, -thermal analysis. -pH-sensitive release kinetics Bone marrow stromal cell culture: -biocompatibility, -osteogenic effects -viability assays, -gene expression analysis, -collagen biomineralization Macrophage interactions were assessed using RAW264.7 cells.	Nanocapsules released 2–7 times more content at acidic pH (3.5) compared to normal pH (7.4). Cell tests showed no toxicity up to 1280 μg/mL and increased bone formation markers. Immune cells absorbed the nanocapsules within 6 h and became anti-inflammatory.	Hollow mesoporous zirconia nanocapsules provide effective pH-responsive delivery of biomineralization precursor, biocompatibility and osteogenic activity.
Pae [58]	To assess the cellular attachment, proliferation, and differentiation of bone marrow–derived osteoblasts grown on smooth zirconia surfaces compared with those coated with calcium phosphate (CaP) or hydroxyapatite (HA), in order to assess their suitability for dental implant applications.	Y-TZP zirconia discs (10 mm diameter, 2 mm thickness) were prepared in three groups: smooth surface (ZS), calcium phosphate coating via ion beam assisted deposition (CaP), and hydroxyapatite coating via aerosol deposition (HA). Bone marrow-derived osteoblasts from rats were grown on the surfaces. Characterization: -MTT (cell proliferation), -SEM, -ALP activity -XPS, -RT-PCR -coatings dissolution in physiological saline.	No significant differences observed between groups in cell proliferation (MTT assay, *p* > 0.05), cell morphology (triangular/spread cells with filopodia), ALP activity (highest in CaP group but *p* > 0.05), or gene expression levels. CaP coatings showed higher Ca^2+^ and P^−^ dissolution rates compared to HA coatings.	HA coating demonstrated superior over time stability compared to CaP coating due to lower dissolution rates.
Faria [56]	To develop a novel zirconia implant design featuring an integrated bioactive composite outer layer to overcome the major limitation of coating detachment in current implants.	Three ceramic powders were used: yttria-stabilized zirconia, HA, and β-TCP. Cylindrical samples were fabricated via press-and-sintering (200 MPa, 1500 °C), creating three groups: -pure zirconia (Z100), -zirconia with 10 vol% HAp outer layer (Z10HAp), -zirconia with 10 vol% β-TCP outer layer (Z10β-TCP). The bioactive outer layers were ~100 µm thick. Characterization: -microstructural analysis, -mechanical properties evaluation, -fatigue testing up to 10^6^ cycles, -surface characterization, -bioactivity assessment through SBF	Gradated zirconia samples with ~100 µm bioactive outer layers showed no delamination and good integration. Composite samples had reduced flexural strength (506–545 vs. 1096 MPa) and fatigue limits (324–346 vs. 859 MPa) but improved fracture toughness.	Study developed a design with integrated bioactive outer layers which eliminates coating detachment issues. Samples met ISO implant standards, had improved fracture toughness.
Hirano [63]	To investigate whether slurry processing could be used to create hydroxyapatite (HAp)-containing surfaces on YSZ (yttria-stabilized zirconia) implants, in order to enhance bone cell activity.	3 mol% yttria-stabilized zirconia (YSZ) discs were coated with a hydroxyapatite (HAp)-forming layer via slurry processing using tribasic calcium phosphate and distilled water, followed by heating at 923 to 1373 K for 2 h. Characterization of -surface morphology, -composition, -HAp thickness Using: -X-ray photoelectron spectroscopy (XPS), -field emission scanning electron microscopy (FESEM), -wavelength dispersive X-ray fluorescence (WDXRF). Bioactivity assessed by: -calcium phosphate precipitation Saos-2 osteoblast-like cell culture: -adhesion, -proliferation, -mineralization	Slurry-treated YSZ surfaces showed temperature-dependent changes, with optimal HAp formation at. Temperature of 1223 K increased calcium phosphate deposition, while 1373 K led to TCP and CaO formation. HAp precipitation in simulated body fluid was highest on the 1223 K surface. The slurry-treated surfaces significantly enhanced calcium deposition during cell differentiation, indicating improved osteogenic potential.	The method effectively deposited HAp on YSZ, enhancing osteogenic activity without affecting cell viability.
Teng [59]	To examine the response of bone tissue to zirconia implants that have been improved with a calcium phosphate coating infused with BMP-2.	Zirconia implants were categorized into three distinct groups: -uncoated (control), -CaP-coated -CaP-coated with BMP2. Coatings were applied using a mineralization process. Characterization: -SEM, -x-ray diffraction (XRD), -ELISA. In total 18 implants were inserted in the mandibles of six beagle dogs after premolar extraction. After three months, histological analysis was performed to assess: -bone volume, -bone-to-implant contact, -marginal bone loss.	-strong primary stability upon insertion -at 3 months notably elevated peri-implant bone volume (%BV) in the CaP with the BMP2 coated group compared to the control group. The CaP-coated group had a slight, not significant increase in bone-to-implant contact compared to the control. Marginal bone loss was lowest in the CaP and BMP2 coated group, though the difference was not significant.	CaP coating with BMP-2 improved bone formation around zirconia implants and showed potential to reduce marginal bone loss.
Stefanic [70]	Development of a novel synthesis method of the thin β-tricalcium phosphate (β-TCP) coating on zirconia implants	Step I: rapid wet-chemical deposition of a biomimetic CaP coating Step II: post-deposition processing -heat treatment at 900 °C -short sonication in a water bath Characterization: -SEM/EDS -XDR -surface roughness -scratch test -tensile bond test -SBF test -adsorption of serum proteins	β-TCP coating characterization: -uniform and dense morphology -thickness of ≈500 nm -roughness in the nanometer range (*R*a = 28 nm) -apatite-mineralization ability in SBF -enhancement of the serum proteins adsorption of on the zirconia. - firm adherence to the zirconia -significant scratch resistance (*L*c = 97 N) -notable tensile strength (52 MPa) - significant resistance to mechanical forces encountered during the implantation process into the artificial bone - modifications to the heating protocol enable enhanced regulation of the topography and, potentially, the physico-chemical characteristics	Presented β-TCP coating turned out to be better in all examined aspects than control and can be used for prospective biomedical applications.
Desante [54]	Multifunctionalities of the inert zirconium dioxide (ZrO2) implant surface by coating it with biomimetic calcium phosphate (CaP) combined with antibiotic-loaded nanoparticles to enhance bioactivity and provide antibacterial effect	Nanoparticles were impregnated with two antibiotics (gentamicin/bacitracin) then immobilized in two coating methods: drop-casting and coprecipitation. Examinations conducted: - scanning electron microscopy (SEM), -X-ray diffraction (XRD), -cross-section analyses -in vitro tests with human mesenchymal stem cells (hMSC) and MG-63 osteoblast-like cells -antibacterial activity assessment	-good cytocompatibility - the cell culture study confirmed a homogeneous distribution of the cells. -elevation in alkaline phosphatase activity -antibacterial effect -coating by coprecipitation gave a more homogeneous effect throughout the entire coating	Crystalline morphology with microcavities of CaP coatings is appropriate for accommodating degradable polymeric nanoparticles infused with antibiotics New biomaterials were shown to deliver drugs in a controlled manner.
Chen [71]	Invention of a new, better surface modification method for zirconia implants by combining femtosecond laser and hydroxyapatite (HA) crystals to induce a rough microstructure and calcium phosphate (CP) deposition in order to promote the osseointegration.	Prepared zirconia discs were separated into 3 groups: -control, -femtosecond laser treatment (FL)—titanium sapphire laser generator and a corresponding regenerative amplification system were used -the experimental group (femtosecond laser treatment combined with hydroxyapatite deposition) (FHA)—before lasing, a suspension of pure HA powder was drop casted on every sample. Characterization: - SEM -EDS - surface roughness - surface wettability -functional chemical groups -XDR, -flexural strength, BMSCs cell culture: -adhesion -proliferation	-surface characterization: microgrooves were noticed in the FL and FHA samples, while the FHA group also showed significant deposition of calcium (Ca) and phosphorus (P) on both the groove walls and the inter-groove flat surfaces. -crystal phase analysis: lack of phase transformation in FL and FHA groups - flexural test strength: highest strength in FHA group, then the FL group, control group the lowest; but no significant difference observed -biological characterization: large number of aligned parallel cells adhered to the flat spaces and grooves between them in FL and FHA groups; significantly higher cell adhesion in FHA group	The changes did not harm or modify the crystal structure or flexural strength of the zirconia. Optimizing a rough and bioactive surface can direct cell alignment along microgrooves and promote cytoskeletal elongation.
Sharif [64]	Enhancement of hydroxyl and carboxyl functional groups through chemically grafting L-Serine onto the zirconia surface to promote calcium-phosphate formation (CPF) and better bonding to bone tissue after implantation	-fabrication and sinterization of discs Mg-PSZ at 1500 °C. -hydroxylation of zirconia surfaces via hydrothermal treatment and phosphoric acid treatment -L-Serine grafting: -calcium-phosphate deposition by soaking specimens in SBF for 7 or 21 days. Characterization: -FTIR-ATR, -TGA, -XRD, -BET, -WCA (contact angle), -FE-SEM/EDS -XPS.	-hydrothermal treatment increased surface –OH groups significantly more than acid treatment. -higher L-Serine grafting at 80 °C and pH 9.5 increased surface negative charge and functional group density. -no apatite formed on untreated zirconia but it did on apatite formed on hydroxylated and L-Serine modified surfaces after 21 days. -apatite layers showed improved density and uniformity under these optimized conditions	L-Serine can successfully be grafted to zirconia via covalent bonding, especially under higher pH and temperature. The modified surface enhances wettability, surface charge, and promotes apatite nucleation and growth.

AFM—Atomic Force Microscopy; ALP—Alkaline Phosphatase; ASTM—American Society for Testing and Materials; BA—Bone Area; BAF—Bone Area Fraction; BIC—Bone-to-Implant Contact; BMMSC—Bone Marrow Mesenchymal Stem Cell; BMSCs—Bone Marrow Stem Cells; B3B test—Ball-on-Three-Balls test; CaP—Calcium Phosphate; Ca^2+^—Calcium ion; CDHA—Calcium-Deficient Hydroxyapatite; COLIA1—Collagen Type I Alpha 1; CPP—Calcium Pyrophosphate; CPT—Calcium Phosphate Tribasic; CP—Calcium Phosphate; CPS—Calcium Phosphate Solution; DCPD—Dicalcium Phosphate Dihydrate; EDX—Energy-Dispersive X-ray Spectroscopy; EDS—Energy-Dispersive Spectroscopy; ELISA—Enzyme-Linked Immunosorbent Assay; FA—Fluorapatite; FE-SEM—Field Emission Scanning Electron Microscopy; FESEM—Field Emission Scanning Electron Microscope; FHA—Femtosecond Laser Treatment Combined with Hydroxyapatite Deposition; FIB—Focused Ion Beam; FGF-2—Fibroblast Growth Factor 2; FTIR—Fourier Transform Infrared Spectroscopy; FTIR-ATR—Fourier Transform Infrared Spectroscopy with Attenuated Total Reflection; GI-XRD—Grazing-Incidence X-ray Diffraction; HA—Hydroxyapatite; hMSC—Human Mesenchymal Stem Cell; HOS—Human Osteosarcoma Cells; H&E—Hematoxylin and Eosin; HV—Vickers Hardness; IBAD—Ion Beam-Assisted Deposition; LMWH—Low Molecular Weight Heparin; MSC—Mesenchymal Stem Cell; MTT—3-(4,5-dimethylthiazol-2-yl)-2,5-diphenyltetrazolium bromide assay; NGIA—Near-Grazing Incidence Angle; OCP—Octacalcium Phosphate; PDL—Periodontal Ligament; PDLSC—Periodontal Ligament Stem Cell; BMP-2—Bone Morphogenetic Protein 2; RUNX2—Runt-related Transcription Factor 2; SBF—Simulated Body Fluid; SEM—Scanning Electron Microscopy; SEM/FIB—Scanning Electron Microscopy/Focused Ion Beam; SEM-EDX—Scanning Electron Microscopy with Energy-Dispersive X-ray; STEM-EDX—Scanning Transmission Electron Microscopy with Energy-Dispersive X-ray; TCP—Tricalcium Phosphate; TEM—Transmission Electron Microscopy; TZ—Tetragonal Zirconia; TZCP—Tetragonal Zirconia Calcium Phosphate; WCA—Water Contact Angle; WDXRF—Wavelength-Dispersive X-ray Fluorescence; XPS—X-ray Photoelectron Spectroscopy; XRD—X-ray Diffraction; YSZ—Yttria-Stabilized Zirconia; Y-TZP—Yttria-Stabilized Tetragonal Zirconia Polycrystal; Zr—Zirconia; ZA—Zirconia–Alumina Composite.

### 3.3. Main Study Outcomes

The studies summarized in Table 2 demonstrated that calcium phosphate coatings on zirconia implants can significantly improve biological responses, particularly by enhancing osseointegration and initial bone apposition. However, differences in coating composition, deposition technique, coating stability, and long-term performance highlight the need for further standardized in vivo studies before clinical translation.

#### 3.3.1. Coating Composition, Deposition Method, Thickness

The reviewed studies employed a wide spectrum of calcium phosphate–based coatings on zirconia substrates. Most investigations applied hydroxyapatite (HAp), either alone or in combination with tricalcium phosphate (TCP), while others examined β-TCP [46,47,70] or octacalcium phosphate (OCP) [52]. Several groups developed composite or multifunctional layers, such as HAp/TCP–zirconia hybrid coatings [51,61], silver nanoparticle–enriched CaP coatings [60], antibiotic-loaded biomimetic layers [54], or biofunctional composites incorporating BMP-2 [59] and FGF-2 with low molecular weight heparin [69]. The deposition methods were equally diverse: RF magnetron sputtering [48,49,50], wet powder spraying [51], aerosol or ion beam–assisted deposition [57,58], electrophoretic deposition [55,66], sol–gel methods [18,62], laser-assisted melting [46,47], and biomimetic precipitation in simulated body fluid [52,53,60,69,71]. Coating thicknesses ranged from nanometric layers of 50–200 nm [57] to micrometric coatings of 1–5 µm [18,49,50,52,62], and up to 100 µm in press-and-sintered composites [56,68].

#### 3.3.2. Surface Roughness and Adhesion Strength

Surface roughness values (Ra) ranged widely, from 0.08 µm in sputtered coatings [50] to over 3.5 µm in wet-sprayed TZCP coatings [51]. Biomimetic and laser-assisted methods generated intermediate roughness values around 0.5–1.0 µm [53,57,64,65]. In terms of adhesion, reported bond strengths also varied significantly. The highest tensile adhesion was observed in layered HAp/TCP coatings (~24.5 MPa) [68], while sputtered CaP layers reached ~38 MPa [5]. Other works evaluated adhesion qualitatively, with most reporting stable coatings resistant to scratching or delamination [51,52,70]. Notably, Stefanic [70] reported a striking difference between β-TCP coatings (52.3 MPa) and biomimetic CaP coatings (2.6 MPa), underlining the strong impact of processing parameters on coating stability.

#### 3.3.3. Biological Responses: Cell Proliferation and ALP Activity

More than two-thirds of the studies (19 out of 27) included in vitro cell culture experiments. Osteoblast-like cell proliferation was consistently enhanced on HAp or CaP-coated zirconia compared to uncoated controls [18,53,54,58,61,62,67,68,71]. Similarly, the activity of alkaline phosphatase (ALP), a marker of osteogenic differentiation, was reported to increase in nearly all experiments that measured it [18,50,53,54,58,61,62,67,71]. Multifunctional coatings enriched with biomolecules or nanoparticles (e.g., BMP-2 [59], antibiotics [54], FGF-2 [69]) demonstrated either additive or synergistic effects, further stimulating osteogenic differentiation.

### 3.4. In Vivo Results

#### 3.4.1. In Vivo Osseointegration and Bone-to-Implant Contact (BIC)

Fifteen of the included studies extended their analyses to in vivo models. Across rabbit, rat, goat, and canine models, calcium phosphate–coated zirconia implants consistently showed improved osseointegration compared to uncoated controls. BIC values were significantly increased in several experiments, such as those by AlFarraj et al. (Zr + HAp: 60.3% vs. uncoated Zr: 45.1%) [65], Cheng et al. (55.3% vs. 43.9%) [18], and Mutsuzaki et al. (48.3% vs. 29.7%) [49]. In a canine model, Langhoff et al. demonstrated early bone apposition (BIC 35.4% vs. 22.5% at 2 weeks) [66], while Ferguson et al. reported increased bone ingrowth in HAp-coated porous zirconia scaffolds [55]. Even when long-term differences were reduced, early bone formation was consistently accelerated in the coated groups [53,59,66].

#### 3.4.2. Early vs. Long-Term Outcomes

The majority of in vivo studies demonstrated significant improvements in early osseointegration and bone formation, while long-term outcomes were more variable. For example, Langhoff et al. [66] observed a pronounced increase in BIC at 2 weeks that was less evident at 4 weeks. Similarly, Cruz et al. [53] and Ferguson et al. [55] observed accelerated early bone apposition in CaP-coated groups, although long-term differences became less consistent. Comparable trends were reported by Mutsuzaki et al. [49] and Teng et al. [59], where coatings facilitated early bone formation but did not always maintain a clear advantage over uncoated zirconia in later stages. These findings suggest that CaP coatings may be particularly advantageous during the early healing phase but require further evaluation regarding their long-term stability and biological performance.

**Table 2:** Detailed characteristics of Included Studies.

Study	Coating Composition	Deposition Method	Coating Thickness (µm)	Surface Roughness (Ra, µm)	Adhesion Strength (MPa/Lc)	Cell Proliferation	ALP Activity	BIC (%)
Safi [46]	β-tricalcium phosphate (β-TCP)	Direct laser melting, Nd:YAG (1064 nm), 90 W	Before preparation 40–50 µm After laser preparation 27 µm	n/a	n/a	n/a	n/a	n/a
Safi [47]	β-Tricalcium phosphate (β-TCP)	direct laser melting Nd:YAG (1064 nm)	n/a	n/a	n/a	PDLSCs, BMMSCs, PDL	n/a	n/a
Kozelskaya [48]	Calcium phosphate tribasic (CPT), calcium phosphate monobasic, calcium phosphate dibasic dehydrate (DCPD), calcium pyrophosphate (CPP) and hydroxyapatite (HA).	Radiofrequency (RF) magnetron sputtering on zirconia	n/a	n/a	n/a	MSC, CPT	n/a	n/a
Pardun [50]	Calcium phosphate (CaP) coating	RF magnetron sputtering.	0.4 μm	0.08 μm	~38 MPa	MC3T3-E1 preosteoblasts	measured at day 7 and day 14	n/a
Stefanic [52]	Octacalcium phosphate (OCP) with a lower layer of calcium-deficient hydroxyapatite (CDHA)	Two-step biomimetic deposition in Ca–P solutions (CPS1 and CPS2) at 37 °C	~5 µm after 3 h in CPS2	n/a	Classified as 4B (ASTM D-3359), >95% coating retention after tape test	n/a	n/a	n/a
Pardun [51]	Mixed coating of calcium phosphate and zirconia	Wet powder spraying (WPS) on presintered zirconia, followed by sintering at 1500 °C for 2 h	~22.59 ± 1.87 µm (TZCP coating)	3.54 ± 0.40 µm	Qualitative—no damage or removal in scratch test with pencil hardness 9H and bovine bone	n/a	n/a	n/a
AlFarraj [65]	Hydroxyapatite (HA) coating on pure titanium and zirconium implants	RF sputter deposition system, followed by infrared heat treatment at 650 °C for 30 s	n/a	0.5 µm for both Ti and Zr	n/a	n/a	n/a	Uncoated Ti: 45.5 ± 13.1% Uncoated Zr: 45.1 ± 14.8% HA-coated Ti: 59.8 ± 16.4% HA-coated Zr: 60.3 ± 17.1%
Yang [68]	Layered HA/TCP on zirconia; top layer = HA, middle/transitional layers become TCP-rich after sintering; minor ZrO_2_/Al_2_O_3_ included in inner layers (biphasic CP).	Low-density slip coating deposition + coating–substrate co-sintering (presintered substrate; final co-sinter at 1300 °C/2 h).	100 (trilayer scaffold coating).	n/a	24.5 MPa (ASTM C633 tensile; failure along coating/substrate interface in the successful test).	L929 fibroblasts: RGR > 90%, cytotoxicity grade 1; cells spread and proliferate, comparable to Ti/Al_2_O_3_ controls.	n/a	n/a
Kong [61]	Biphasic calcium phosphate (HA + TCP) formed in situ; composition varied (0–40 vol% HA in ZA matrix).	Powder mixing (ZA nano-composite powder via Pechini process) + hot pressing at 1400 °C, 30 MPa, 1 h	n/a	n/a	n/a	MG63 osteoblast-like cells: proliferation ↑ with HA content; significant increase from 10% HA, max at 40% HA (comparable to pure HA).	HOS cells: ALP ↑ with HA content; significant from 20% HA, max at 40% HA (comparable to pure HA).	n/a
Lee [57]	Calcium phosphate (CaP) nano-layer (Ca/P 1.67) on micro-structured zirconia (ZiUnitet).	Surface A: sequential immersion in phosphorous-rich then calcium-rich solution; Surface C: droplet application of HA nanoparticles + heat treatment.	A: <0.05 µm (50 nm); C: <0.2 µm (200 nm)	ZiUnitet & CaP-modified ZiUnitet: 1.0 µm; TiUnitet: 1.3 µm	n/a	n/a	n/a	3 weeks: A: 64.6 ± 3.6, C: 62.2 ± 3.1, ZiUnitet: 70.5 ± 3.1, TiUnitet: 77.6 ± 2.6; 6 weeks: A: 68.6 ± 1.9, C: 64.5 ± 4.1, ZiUnitet: 69.7 ± 5.7, TiUnitet: 67.1 ± 4.2
Ferguson [55]	Hydroxyapatite (HA) coating on porous zirconia structures.	Electrophoretic deposition (EPD) of HA slurry into porous zirconia scaffolds, followed by sintering.	~30–50 µm (coating in pores and on surface struts)	n/a	n/a	n/a	n/a	Goat model (12 weeks): BIC significantly higher for HA-coated porous zirconia than uncoated (exact % not reported, only histological evidence of increased bone ingrowth)
Kim [62]	Hydroxyapatite (HA)/β-tricalcium phosphate (β-TCP) composite coating on zirconia	Sol–gel-derived CaP coating with heat treatment at 500 °C	~1 µm	n/a	n/a	Rat calvaria osteoblast-like cells: proliferation ↑ over time; higher on HA/β-TCP-coated zirconia than uncoated control	ALP activity ↑ on coated zirconia, peak at 14 days, significantly higher than uncoated	n/a
Langhoff [66]	Calcium phosphate (CaP) coating on zirconia oral implants	Electrophoretic deposition (EPD) followed by heat treatment	n/a	n/a	n/a	n/a	n/a	Mini pig model: BIC at 2 weeks — CaP-coated zirconia: 35.4 ± 7.4, Uncoated zirconia: 22.5 ± 8.5; at 4 weeks — CaP-coated: 46.6 ± 5.9, uncoated: 40.5 ± 10.3
Cheng [18]	Hydroxyapatite (HA) coating on zirconia	Sol–gel dip-coating followed by sintering at 800 °C	~1 µm	Coated: 0.62 ± 0.07; Uncoated: 0.15 ± 0.02	n/a	MC3T3-E1 cells: proliferation ↑ on HA-coated zirconia vs. uncoated (significant from day 3 onward)	ALP activity ↑ on coated zirconia at day 7 and 14 vs. uncoated	Rabbit model, 4 weeks: BIC coated zirconia 55.27 ± 6.12%, uncoated 43.94 ± 4.74%
Mutsuzaki [49]	Low-crystallinity hydroxyapatite (HA) coating on zirconia.	Er:YAG pulsed laser deposition at room temperature	~1 µm	n/a	n/a	n/a	n/a	Rabbit tibia model, 6 weeks: BIC coated zirconia 48.3 ± 17.4%, uncoated 29.7 ± 16.0%
Cruz [53]	Calcium phosphate (CaP) coating on zirconia	Biomimetic deposition from simulated body fluid (SBF), 37 °C, 14 days	n/a	Coated: 0.45 ± 0.08; Uncoated: 0.07 ± 0.02	n/a	Human osteoblast-like SaOs-2 cells: proliferation ↑ with coating maturation time, significantly higher after 14 days deposition vs. uncoated	ALP activity ↑ in coated samples, maximal after 14 days deposition	n/a
Goldschmidt [60]	Calcium phosphate with silver nanoparticles	Biomimetic coating	n/a	n/a	n/a	0.1 g/L Ag-NPs supported MG63 cell growth the rest was cytotoxic	n/a	n/a
Yasuaga [69]	Fibroblast growth factor-2 (FGF-2) + low molecular weight heparin (LMWH) + CaP	Biomimetic precipitation	n/a	n/a	n/a	Higher cell growth in both mouse cells and human umbilical vein endothelial cells cultures in coatings that contained FGF-2 together with LMWH	n/a	n/a
Huang [67]	Poly(allylamine)-stabilized ACP (PAH-ACP)	Mixing hmZrO_2_ (hollow mesoporous zirconia nanoparticles) with PAH-ACP	n/a	n/a	n/a	No significant difference in cell proliferation between control and most PAH-ACP@hmZrO_2_ concentrations, except at the highest tested concentration, where a decrease was observed.	Cells treated with PAH-ACP@hmZrO_2_ showed clearly higher ALP activity	n/a
Pae [58]	CaP group: Calcium phosphate from mixed hydroxyapatite and calcium oxide powders). HA group: Hydroxyapatite coating from aerosol deposition	CaP group: IBAD HA group: aerosol deposition	CaP group: up to 0.5 µm HA group: n/a	n/a	n/a	Similar proliferation in control, CaP, and HA groups. CaP group had the highest value at 4 h, but differences were not significant	After 14 days, ALP activity was increased in CaP, then control and lowest in HA	n/a
Faria [56]	Hydroxyapatite (HAp) or beta-tricalcium phosphate (β-TCP)	Press and sintering technique	100 µm	n/a	Z100 (control) 0.018 ± 0.006 Z10HAp 0.070 ± 0.009 Z10β-TCP 0.108 ± 0.026	n/a	n/a	n/a
Hirano [63]	Tribasic calcium phosphate powder	Zirconia discs buried in calcium-phosphate slurry	n/a	n/a	n/a	Cell number was similar on treated and untreated zirconia surfaces at both 6 h and 72 h, with no significant differences observed.	n/a	n/a
Teng [59]	Amorphous CaP and CaP solution with BMP-2	Biomimetic CaP coating and biomimetic CaP coating with incorporated BMP-2	50 μm	n/a	n/a	n/a	n/a	%BIC was somewhat elevated both in the CaP group and CaP + BMP-2 group than in the control
Stefanic [70]	β-tricalcium phosphate coating on zirconia implants	biomimetic CaP coating: a 2-step immersion in solutions of varying pH β-TCP coating: heat treatment at 900 ◦C, sonic treatment in water	12 µm	28 ± 2 nm	Tensile strength test (MPa): 52.3 ± 3.8 for β-TCP 2.6 ± 0.4 for biomimetic CaP coating	n/a	n/a	n/a
Desante [54]	Calcium phosphate coating doped with nanoparticles encapsulating antibiotics	Immersion of Zirconia substrates in this mixture of CaP. Next immersion in similar mixture but without Mg^2+^ and HCO_3_^−^ Prepared nanoparticles with antibiotics were added by methods of coprecipitation and drop casting	First coating: thinner than 2 µm Second coating: approximately double the first coating’s thickness, ~4 µm	Exact Ra values not given Uncoated-low Ra due to polishing First coating: Ra increased Second coating: Ra decreased by ~⅔, smoother but still rougher-than-uncoated surface.	n/a	MG-63 -mostly viable, -spindle shape, -adhered and proliferated well hMSC -mostly viable, -well-spread, -even distribution, -increasing adhesion over time	Higher ALP on CaP-coated samples highest on samples with drug loaded nanoparticles	n/a
Chen [71]	Femtosecond laser cured hydroxyapatite coating on yttria stabilized tetragonal zirconia	Drop casting then treatment with femtosecond laser	n/a	Micro-grooves were observed in laser treated group and laser treated with hydroxyapatite (FHA) group; granular material deposits in the grooves of the FHA group; groove width ≈15 µm depth ≈6 µm For FHA: Sa: 1.01 ± (0.09 b) µm Sq: 1.41 ± (0.14 d) µm Sz: 5.59 ± (0.32) µm	n/a	In both groups treated with laser numerous cells were observed adhering to the grooves and the flat regions in between compared to control group where proliferation was sparse. Laser in combination with hydroxyapatite had the best results.	FHA—enhanced ALP activity, but no numerical values or units provided.	n/a
Sharif [64]	L-serine–functionalized Mg-partially stabilized zirconia (Mg-PSZ) surface with in vitro–formed calcium-phosphate (hydroxyapatite) layer	1. Hydrothermal hydroxylation of Mg-PSZ (120 °C, 6 or 40 h) 2. Chemical grafting of L-serine in aqueous solution (0.01 mg/mL, 40–80 °C, 15–60 min, pH 5.5 or 9.5) 3. Immersion in 1.5 × SBF (pH 7.4, 37 °C) for 7 or 21 days to deposit apatite	n/a Only particle/cluster sizes are given: ~6–7 µm after 21 days SBF; ~3–3.2 µm after 7 days at pH 9.5	n/a n/a	n/a n/a	n/a	n/a	n/a

ACP—amorphous calcium phosphate; Ag-NPs—silver nanoparticles; ALP—alkaline phosphatase; Al_2_O_3_—alumina; ASTM—American Society for Testing and Materials; BIC—bone-to-implant contact; BMMSCs—bone marrow mesenchymal stem cells; BMP-2—bone morphogenetic protein 2; CP—calcium phosphate; CPT—calcium phosphate tribasic; CPP—calcium pyrophosphate; DCPD—dicalcium phosphate dihydrate; EPD—electrophoretic deposition; Er:YAG-PLD—erbium:YAG pulsed laser deposition; FA—fluorapatite; FHA—femtosecond laser + hydroxyapatite; HA—hydroxyapatite; HA/TCP—hydroxyapatite/tricalcium phosphate; hmZrO_2_—hollow mesoporous zirconia; HOS—human osteosarcoma cells; IBAD—ion beam–assisted deposition; L929—mouse fibroblast cell line L929; Lc—critical load; MC3T3-E1—mouse pre-osteoblast cell line; Mg-PSZ—magnesia partially stabilized zirconia; MPa—megapascal; MSC—mesenchymal stem cell; n/a—not applicable; Nd:YAG—neodymium-doped yttrium aluminum garnet (laser); nm—nanometer; OCP—octacalcium phosphate; PAH-ACP—poly(allylamine)-stabilized amorphous calcium phosphate; PDL—periodontal ligament; PDLSCs—periodontal ligament stem cells; Ra—arithmetic mean roughness; RGR—relative growth rate; RF—radiofrequency; Sa—area roughness parameter; SBF—simulated body fluid; Sq—root-mean-square surface height; Sz—maximum height of surface; TCP—tricalcium phosphate; Ti—titanium; TZCP—tetragonal zirconia calcium phosphate; µm—micrometer; WPS—wet powder spraying; Y-TZP—yttria-stabilized tetragonal zirconia polycrystal; ZA—zirconia–alumina composite; Zr—zirconia; ZrO_2_—zirconium dioxide.

### 3.5. Quality Assessment of Included Studies

Across all questions, 12 papers achieved the maximum score, providing positive responses to all nine items [49,53,54,55,57,58,59,60,66,69,70,71], 4 papers scored 8 points [46,56,65,67], 6 papers scored 7 points [47,48,50,51,61,63] and 5 papers received a favorable response to 6 of them [18,52,62,64,68] (see Figure 4 and Supplementary Table S1).

## 4. Discussion

This review evaluates the role of calcium phosphate coatings in enhancing zirconia implant osseointegration. Its primary objective is to critically and systematically evaluate the available evidence concerning such surface modifications presented in the existing literature [12,72,73]. A comprehensive analysis reveals compelling findings from numerous studies indicating that the use of calcium phosphate coatings on zirconia implants enhances cell proliferation [18,47,48,50,53,54,58,60,61,62,68,69,71], increases alkaline phosphatase (ALP) activity [18,50,53,54,58,61,62,67,71] and improves osseointegration as well as bone-to-implant contact (BIC) [18,49,55,57,59,65,66]. Zirconia’s relatively low intrinsic bioactivity can limit the predictability of osseointegration compared with more reactive implant materials. This characteristic highlights the critical role of bioactive surface modifications, such as calcium phosphate coatings, in promoting cellular adhesion, osteoblast proliferation, and early bone formation. By creating a more favorable interface between the implant and adjacent bone, these coatings can enhance both the quality and consistency of zirconia implant integration [10,11]. Despite increasing research on this topic, no systematic review has yet comprehensively evaluated the results of calcium phosphate coatings specifically on zirconia implants. The present work aims to address this gap by synthesizing and critically assessing the available evidence, providing a clearer understanding of their potential to improve zirconia implant performance. A broad range of deposition techniques was employed, including biomimetic precipitation as one of the most common approaches, with some studies incorporating additional bioactive compounds such as silver nanoparticles, low-molecular-weight heparin combined with fibroblast growth factor-2, or nanoparticles encapsulating antibiotics to further enhance coating performance.

In the included studies, a wide range of calcium phosphate derivatives was employed as coating materials. Simpler salts such as monobasic, dicalcium (including the dihydrate form), and tribasic calcium phosphate, as well as β-tricalcium phosphate, were reported [74,75]. More complex phases, including hydroxyapatite, calcium-deficient hydroxyapatite, octacalcium phosphate, calcium pyrophosphate, and poly(allylamine)-stabilized amorphous calcium phosphate, were also investigated [76,77]. In several studies, these compounds were additionally applied in nanoparticulate form, reflecting the diversity of calcium phosphate–based strategies explored for implant modification. This heterogeneity likely results from differences in solubility, resorption behavior, and biological responses, which may influence their clinical applicability [78]. A consistent trend was observed among studies assessing ALP activity: calcium phosphate coatings generally produced a notable increase in ALP levels, particularly over longer observation periods [79,80]. In the study by Pae et al., a direct comparison between CaP and HA revealed superior results for CaP coatings, likely due to the higher bioavailability of simpler CaP particles compared to the more complex HA structure [58]. Nevertheless, hydroxyapatite coatings also yielded favorable outcomes in other studies [18,81]. Regarding cell proliferation, most studies demonstrated a promoting effect of calcium phosphate coatings, although a few, including those by Huang et al., Pae et al., and Hirano et al., reported no statistically notable distinctions in relation to control groups [58,63,67]. These differences highlight the need for further investigation and more standardized experimental designs to determine the full extent of calcium phosphate coating efficacy.

Significant variability exists in the methodologies used to fabricate implant coatings, as differences in physical forces, chemical factors, and preparation techniques inevitably influence the results [82]. Consequently, selecting an appropriate coating method depends heavily on the objectives of each study and the desired biological outcome [83,84,85]. A broad range of deposition techniques was employed, including biomimetic precipitation- as one of the most common approaches, with some studies incorporating additional bioactive compounds such as silver nanoparticles, low-molecular-weight heparin combined with fibroblast growth factor-2 or nanoparticles encapsulating antibiotics to enhance coating performance [52,53,54,60,69]. One of the few directly comparable parameters was coating thickness. For example, Stefanic et al. reported a layer thickness of approximately 5 µm, while Desante et al. achieved 2–4 µm [52,54]. Conversely, the highest coating thicknesses were documented by Yang et al. and Faria et al., reaching up to 100 µm, both using HA/TCP composites but employing distinct fabrication methods [56]. In the first approach, the outer layer was decomposed into TCP during processing, while in the second, a bioactive composite was added following heat treatment. Although these findings collectively indicate promising outcomes, the significant heterogeneity of methodologies complicates the development of standardized guidelines.

A notable trend emerging from the literature is the positive correlation between the simplicity of coating compounds and the resulting levels of ALP activity [18,48]. However, a considerable number of studies failed to report fundamental physical parameters, including surface roughness, coating thickness, and adhesion strength [29,51,62,86]. The lack of consistent and comparable data on key physical parameters currently prevents the identification of the most effective coating technique [58]. Moreover, due to substantial heterogeneity in coating compositions and fabrication methods, no clear consensus can be reached regarding which calcium phosphate derivative provides the most favorable biological response [51,52,55,67]. This omission severely limits the ability to compare findings across studies. Consequently, the existing body of evidence does not yet support definitive recommendations regarding optimal coating strategies. To achieve more reliable conclusions, future research should focus on well-designed experiments with standardized protocols and consistent reporting of both biological and physical characteristics.

Several important limitations should be taken into account when interpreting these results. The predominance of short-term studies with limited follow-up does not allow for a reliable assessment of the long-term stability of calcium phosphate coatings under physiological loading conditions, which is essential for clinical applications [49,85]. Although in vitro studies consistently demonstrate enhanced cell proliferation, ALP activity, and bone-to-implant contact, the translation of these findings into predictable clinical success remains uncertain, as simplified laboratory settings may not adequately reflect the complexity of the in vivo environment [48]. Moreover, critical aspects such as coating durability under functional loading, scalability of production, and commercial feasibility remain largely unresolved [29,50,51]. While calcium phosphate coatings have been successfully applied to titanium, no long-term randomized clinical trials have yet confirmed superior outcomes for coated zirconia implants [85,87]. Therefore, although biologically promising, CaP coatings on zirconia should currently be regarded as an investigational approach until validated by robust clinical evidence supported by standardized protocols and extended follow-up.

## 5. Conclusions

Based on the gathered evidence on cell proliferation and bioactivity, calcium phosphate coatings represent a highly promising strategy for modifying zirconia implant surfaces to reduce their inherent inertness and promote osseointegration. Most studies report beneficial effects on cell adhesion, differentiation and proliferation, as well as increased alkaline phosphatase activity and improved bone-to-implant contact, suggesting that calcium phosphate layers can significantly enhance biological integration. Future research should prioritize the standardization of experimental protocols, including precise reporting of coating characteristics, biological testing parameters, and long-term performance. Comparative studies between different calcium phosphate compounds—particularly simpler salts versus complex phases such as hydroxyapatite—are needed to establish optimal formulations. Furthermore, extending investigations into in vivo studies and clinical trials will be crucial to confirm the translational relevance of these findings. Despite these limitations, the current evidence indicates that calcium phosphate coatings hold strong potential to improve the clinical success of zirconia implants and may become an integral component of future implant surface engineering strategies.

## Supplementary Materials

The following supporting information can be downloaded at: https://www.mdpi.com/article/10.3390/ma18194501/s1, Table S1: Quality assessment of included studies.
