# Supplementary material for: Enhancing Osseointegration of Zirconia Implants Using Calcium Phosphate Coatings: A Systematic Review
Source: Materials (Basel). 2025 Sep 27;18(19):4501. doi: 10.3390/ma18194501 (PMC12525974; doi:10.3390/ma18194501)
Supplement: Supplementary file 1 [file materials-18-04501-s001.zip › materials-3865505-supplementary.pdf]

This table presents the Joanna Briggs Institute (JBI) checklist for quasi-experimental studies, applied in the systematic review titled: **Enhancing Osseointegration of Zirconia Implants Using Calcium Phosphate Coatings: A Systematic Review**

Jacek Matys [1,*], Ryszard Rygus [2], Julia Kensy [3], Krystyna Okoniewska [4], Wojciech Zakrzewski [5], Agnieszka Kotela [5], Natalia Struzik [6], Hana Gerber [7], Magdalena Fast [8] and Maciej Dobrzyński [9,*]

[1] Dental Surgery Department, Wroclaw Medical University, Krakowska 26, 50-425 Wroclaw, Poland; jacek.matys@umw.edu.pl
[2] Hospital in Świdnica, Leśna 27/29, 58-100 Świdnica, Poland; ryszard.w.rygus@gmail.com
[3] Faculty of Dentistry, Wroclaw Medical University, Krakowska 26, 50-425 Wroclaw, Poland; julia.kensy@student.umw.edu.pl
[4] University hospital in Wroclaw, Borowska 213, 50-556 Wroclaw, Poland; krystyna.okoniewska@vet-farma.com.pl
[5] Medical Center of Innovation, Wroclaw Medical University, Krakowska 26, 50-425 Wroclaw, Poland; kotela.agnieszka@gmail.com; wojciech.zakrzewski1992@gmail.com
[6] Pre-Clinical Research Centre, Wroclaw Medical University, Bujwida 44, 50-368 Wroclaw, Poland; natalia.struzik98@gmail.com
[7] Department of Maxillofacial Surgery, Wroclaw Medical University, 50-556 Wrocław, Poland; hanna.gerber@umw.edu.pl
[8] Department of Drug Form Technology, Wroclaw Medical University, Borowska 211 A, 50-556 Wroclaw, Poland; magdalena.fast@umw.edu.pl
[9] Department of Pediatric Dentistry and Preclinical Dentistry, Wroclaw Medical University, Krakowska 26, 50-425 Wroclaw, Poland; maciej.dobrzynski@umw.edu.pl

* Correspondence: jacek.matys@umw.edu.pl; maciej.dobrzynski@umw.edu.pl

Supplementary Table S1. Quality assessment of Included studies.

Authors	1. Is It Clear in the Study What Is the 'Cause' and What Is the 'Effect'?	2. Were the Participants Included in Any Comparisons Similar?	3. Were the Participants Included in Any Comparisons Receiving Similar Treatment/Care, Other than the Exposure or Intervention of Interest?	4. Was There a Control Group?	5. Were There Multiple Measurements of the Outcome Both Pre and Post the Intervention/Exposure?	6. Was Follow up Complete and If Not, Were Differences Between Groups in Terms of Their Follow up Adequately Described and Analyzed?	7. Were the Outcomes of Participants Included in Any Comparisons Measured in the Same Way?	8. Were Outcomes Measured in a Reliable Way?	9. Was Appropriate Statistical Analysis Used?
Safi [79]	Yes	Yes	Yes	Yes	Yes	Yes	Yes	Yes	No
Safi [80]	Yes	Yes	No	Yes	Yes	Yes	Yes	Yes	No

Kozelskaya [81]	Yes	Yes	Yes	No	No	Yes	Yes	Yes	Yes
Pardun [83]	Yes	Yes	Yes	No	No	Yes	Yes	Yes	Yes
Stefanic [85]	Yes	Yes	Yes	No	No	Yes	Yes	Yes	No
Pardun [84]	Yes	Yes	Yes	No	No	Yes	Yes	Yes	Yes
AlFarraj [98]	Yes	Yes	Yes	Yes	Yes	Yes	Yes	Yes	Yes
Yang [101]	Yes	Yes	Yes	No	No	Yes	Yes	Yes	No
Kong [94]	Yes	Yes	Yes	No	No	Yes	Yes	Yes	Yes
Lee [90]	Yes	Yes	Yes	Yes	Yes	Yes	Yes	Yes	Yes
Ferguson [88]	Yes	Yes	Yes	Yes	Yes	Yes	Yes	Yes	Yes
Kim [95]	Yes	Yes	Yes	No	No	Yes	Yes	Yes	No
Langhoff [99]	Yes	Yes	Yes	Yes	Yes	Yes	Yes	Yes	Yes
Cheng [32]	Yes	Yes	Yes	No	No	Yes	Yes	Yes	No
Mutsuzaki [82]	Yes	Yes	Yes	Yes	Yes	Yes	Yes	Yes	Yes
Cruz [86]	Yes	Yes	Yes	Yes	Yes	Yes	Yes	Yes	Yes

Goldschmidt [93]	Yes	Yes	Yes	Yes	Yes	Yes	Yes	Yes	No
Yasuaga [102]	Yes	Yes	Yes	Yes	Yes	Yes	Yes	Yes	Yes
Huang [100]	Yes	Yes	Yes	Yes	No	Yes	Yes	Yes	Yes
Pae [91]	Yes	Yes	Yes	Yes	Yes	Yes	Yes	Yes	Yes
Faria [89]	Yes	Yes	Yes	Yes	Yes	Yes	Yes	Yes	No
Hirano [96]	Yes	Yes	Yes	No	No	Yes	Yes	Yes	Yes
Teng [92]	Yes	Yes	Yes	Yes	Yes	Yes	Yes	Yes	Yes
Stefanic [103]	Yes	Yes	Yes	Yes	Yes	Yes	Yes	Yes	Yes
Desante [87]	Yes	Yes	Yes	Yes	Yes	Yes	Yes	Yes	Yes
Chen [104]	Yes	Yes	Yes	Yes	Yes	Yes	Yes	Yes	Yes
Safi [79]	Yes	Yes	Yes	Yes	No	No	Yes	Yes	No
